# Performance of the cross-polarization experiment in conditions of radiofrequency field inhomogeneity and slow to ultrafast magic angle spinning (MAS)

**DOI:** 10.5194/mr-4-199-2023

**Published:** 2023-08-15

**Authors:** Andrej Šmelko, Jan Blahut, Bernd Reif, Zdeněk Tošner

**Affiliations:** 1 Department of Chemistry, Faculty of Science, Charles University, Albertov 6, 12842 Prague, Czech Republic; 2 Institute of Organic Chemistry and Biochemistry of the CAS, Flemingovo náměstí 2, 16610, Prague, Czech Republic; 3 Bayerisches NMR Zentrum (BNMRZ) at School of Natural Sciences, Department of Bioscience, Technische Universität München (TUM), Lichtenbergstr. 4, 85747 Garching, Germany; 4 Helmholtz-Zentrum München (HMGU), Deutsches Forschungszentrum für Gesundheit und Umwelt, 85764 Neuherberg, Germany

## Abstract

In this paper, we provide an analytical description of the performance of the cross-polarization (CP) experiment, including linear ramps and adiabatic tangential sweeps, using effective Hamiltonians and simple rotations in 3D space. It is shown that radiofrequency field inhomogeneity induces a reduction in the transfer efficiency at increasing magic angle spinning (MAS) frequencies for both the ramp and the adiabatic CP experiments. The effect depends on the ratio of the dipolar coupling constant and the sample rotation frequency. In particular, our simulations show that for small dipolar couplings (1 kHz ) and ultrafast MAS (above 100 kHz ) the transfer efficiency is below 40 % when extended contact times up to 20 ms are used and relaxation losses are ignored. New recoupling and magnetization transfer techniques that are designed explicitly to account for inhomogeneous radiofrequency fields are needed.

## 1 Introduction

Cross-polarization (CP) is a remarkable experiment with a very long history (Schaefer, 2007). Hartmann and Hahn (1962) presented the theory of magnetization transfer in a two-spin system under conditions of double radiofrequency (RF) irradiation of a static sample. Pines et al. (1973) published their seminal work on proton-enhanced solid-state NMR of dilute spins such as ${}^{13}C$ and ${}^{15}N$ . While magic angle spinning (MAS) was already introduced by Andrew et al. (1958) and independently by Lowe (1959), it was only in 1977 that cross-polarization was successfully combined with sample rotation. The necessary modification of the Hartmann–Hahn conditions was described in Stejskal et al. (1977). After that, many modifications with variable amplitude irradiations on one or both RF channels were developed. Among them, simple linear ramps (Metz et al., 1994) and adiabatic sweeps (Hediger et al., 1995) became the most popular. Ramp CP was originally introduced to broaden the Hartmann–Hahn (HH) matching condition and to obtain uniform signal amplitudes. In the original publication, low MAS frequencies (below ∼ 10 kHz ) were used, and the sweep could cover several HH conditions. At the same time, it was realized that the largest enhancement in signal intensity is obtained when the sweep covers only one HH condition (Metz et al., 1994). The RF amplitude sweep implies a partially adiabatic inversion of the spins and compensates for RF field inhomogeneities (Peersen et al., 1994; Hediger et al., 1995).

Until now, cross-polarization remains the main pulse sequence building block for magnetization transfers. At very high MAS frequencies, it becomes difficult to achieve HH zero-quantum matching where the difference between the two applied RF amplitudes is equal to the MAS frequency. Instead, the HH double-quantum matching condition must be used, in which the sum of the RF amplitudes equals the MAS frequency. The spin dynamics remain the same with the exception that negative intensities are obtained (Meier, 1992). Cross-polarization is thus applied over an exceptionally wide range of conditions: from experiments using static samples to MAS experiments with rotation frequencies above 100 kHz .

The most widespread coil design used by all vendors in most of the MAS solid-state NMR probes is a solenoid. Its simple design, large filling factor, high conversion ratio from RF power to RF field and its possibility of being integrated into circuits tuned to multiple frequencies are among the major benefits. The main drawback is its inhomogeneous RF field, which quickly decays towards the end of the coil, where the RF amplitude is reduced to about 50 % of the value achieved in the coil center. Several other strategies have been proposed to design NMR coils that are compatible with MAS and provide improved RF field homogeneity. Variable pitch coils were proposed by Idziak and Haeberlen (1982) and recently explored by Martin and coworkers, who proposed 3D-printed templates for easy manufacturing (Kelz et al., 2019). An interesting alternative was proposed by Privalov et al. (1996) using variable ribbon-width coils that improve RF homogeneity not only along the coil axis but also in the radial direction. Another type of coil was designed for so-called E-free probes, which minimize sample heating effects induced by high-power RF irradiation. These coils also show improved RF field homogeneity (Krahn et al., 2008). All strategies have benefits and disadvantages. Variable-pitch coils provide a lower RF conversion ratio and thus lower sensitivity. E-free probes consist of separated coils for the high- and low-frequency RF channels, which potentially leads to different RF field profiles and imbalances between these channels. Worth mentioning is the recent cryo-CP-MAS probe technology that is reported to provide excellent RF field homogeneity (Hassan et al., 2020).

RF field inhomogeneity is a concern for the performance of virtually all NMR experiments. Specifically, it affects the sensitivity of the cross-polarization experiment, since the Hartmann–Hahn matching is violated at different positions within the sample as a consequence of the modulation of the RF amplitudes due to inhomogeneity. An experimental example of this volume-selective behavior of the cross-polarization experiment is presented in the work of Tošner et al. (2018). In biomolecular applications, it is difficult to prepare large quantities of isotopically labeled samples, and only limited amounts of material are available that do not allow us to completely fill the MAS rotor. To yield the highest possible sensitivity, samples are typically packed around the center of the coil, and the problem of RF field distribution is reduced. However, the rotors for ultrafast MAS are small and can be completely filled with sample. Under these conditions, RF inhomogeneity comes up as a concern in its full range. With faster MAS and correspondingly smaller rotors that contain less material, we again face sensitivity issues. It is obviously desirable that the whole sample contributes to the NMR signal. At this point, it appears that the inhomogeneity of the RF field is the prevailing challenge for the development of new solid-state NMR methods.

In this tutorial article, we summarize the principles of the cross-polarization (CP) experiment and focus on the effect of RF field inhomogeneity. For demonstration purposes we limit our treatment to an isolated heteronuclear pair of spin- 1/2 nuclei that are coupled via the dipole–dipole interaction. We assume that there is no chemical shift interaction. Using average Hamiltonian theory and simple 3D rotations we explain the process of magnetization transfer assuming different amplitude-swept CP variants. We show that the total signal measured after the CP transfer decreases with increasing MAS frequency. The effect is amplified for small dipolar couplings. We numerically optimize linear ramp and adiabatic tangential sweep experiments to identify the conditions for the best performance as a function of the dipolar coupling constant, contact time and MAS frequency. Neither of these techniques under any condition fully compensates for RF field inhomogeneities. The most striking example of low efficiency is the CP transfer between a ${}^{15}N$ nucleus directly bonded to a ${}^{13}C$ atom involving a dipolar coupling constant of about 1 kHz . With the forthcoming MAS technology in mind that can reach MAS frequencies of up to 200 kHz , we predict that only 20 % of the sample will contribute to the NMR signal after a CP mixing time of 10 ms . It clearly calls for the development of alternative magnetization transfer techniques that are suitable for ultrafast MAS NMR experiments.

## 2 Theory

A theoretical description of the cross-polarization phenomenon can be found in many solid-state NMR textbooks. Here, we revisit the relevant parts and focus on visualization of the magnetization transfer process during variable-amplitude sequences, following the description presented by Rovnyak (2008). In the following, we assume an isolated spin pair. A more general description that considers the surrounding spins and homonuclear interactions within an $I_{N}S$ spin system can be found, for example, in the work of Vega and coworkers (Marks and Vega, 1996; Ray et al., 1998). This issue has been reviewed in the context of ultrafast MAS by Emsley and coworkers (Laage et al., 2009), concluding that the perturbation effects of homonuclear interactions diminish with increasing spinning rate. The authors infer that the behavior of the CP experiment at very fast spinning in a $I_{N}S$ spin system is reminiscent of a ${}^{13}C$ – ${}^{15}N$ spin pair, which we would like to analyze in the following in detail.

### 2.1 Hamiltonian decomposition into zero-quantum (ZQ) and double-quantum (DQ) subspaces

We start with the Hamiltonian that contains the dipole–dipole interaction and the radiofrequency fields with amplitudes $\omega_{I}$ and $\omega_{S}$ applied on resonance to spins I and S , respectively.

1 $$H=\omega_{I}I_{x}+\omega_{S}S_{x}+d_{\text{IS}}(t)2I_{z}S_{z}$$

The dipolar term is time dependent due to magic angle spinning (angular frequency $\omega_{R}$ ) and can be expressed as

2dIS(t)=g1cos(ωRt+γ)+g2cos(2ωRt+2γ),3g1=-122πbISsin(2β),4g2=122πbISsin2(β),

where $b_{\text{IS}}$ is the dipolar coupling constant ( $b_{\text{IS}}=-\frac{\mu }{4\pi }\frac{\gamma_{I}\gamma_{S}\hbar }{r_{\text{IS}}^{3}}\frac{1}{2\pi }$ ) in units of hertz and β and γ are the Euler angles relating the orientation of the dipolar vector $r_{\text{IS}}$ to the rotor axis (the α angle is irrelevant as the dipolar coupling tensor has a vanishing asymmetry).

Subsequently, the reference frame is transformed into the tilted frame where the radiofrequency fields are linear with $I_{z}$ and $S_{z}$ , while the dipolar term becomes transversal. This transformation is represented by a π/2 rotation around $(I_{y}+S_{y}$ ) and we obtain

5 $$H^{\prime }=\omega_{I}I_{z}+\omega_{S}S_{z}+d_{\text{IS}}(t)2I_{x}S_{x}.$$

**Table 1 Ch1.T1:** Fictitious spin- 1/2 operators in zero-quantum and double-quantum subspaces.

Zero quantum	Double quantum	
$I_{x}^{\text{ZQ}}=I_{x}S_{x}+I_{y}S_{y}$	$I_{x}^{\text{DQ}}=I_{x}S_{x}-I_{y}S_{y}$	
$I_{y}^{\text{ZQ}}=I_{y}S_{x}-I_{x}S_{y}$	$I_{y}^{\text{DQ}}=I_{y}S_{x}+I_{x}S_{y}$	
$I_{z}^{\text{ZQ}}=\frac{1}{2}(I_{z}-S_{z})$	$I_{z}^{\text{DQ}}=\frac{1}{2}(I_{z}+S_{z})$	
Inverted relations		
$I_{z}=I_{z}^{\text{DQ}}+I_{z}^{\text{ZQ}}$	$S_{z}=I_{z}^{\text{DQ}}-I_{z}^{\text{ZQ}}$	$2I_{x}S_{x}=I_{x}^{\text{DQ}}+I_{x}^{\text{ZQ}}$

This form of the Hamiltonian allows decomposition of the spin dynamics problem into two separate subspaces, the zero-quantum (ZQ) and the double-quantum (DQ) subspace. The ZQ and DQ subspaces can be represented using fictitious spin- 1/2 operators that are defined in Table [Table Ch1.T1].

The Hamiltonian can then be written as

6H′=HZQ+HDQ,7HZQ=ωI-ωSIzZQ+dIS(t)IxZQ,8HDQ=ωI+ωSIzDQ+dIS(t)IxDQ.

### 2.2 Magnetization transfer in the static CP experiment

The magnetization transfer process in the tilted frame is described by a transition from $I_{z}$ into $S_{z}$ . The action of RF pulses and the dipolar interaction on the spin state $I_{z}$ in the tilted frame are evaluated independently in the ZQ and DQ subspace, working with the initial spin states $I_{z}^{\text{ZQ}}$ and $I_{z}^{\text{DQ}}$ , respectively. If the sample is static, the zero-quantum Hartmann–Hahn condition is $\omega_{I}-\omega_{S}=0$ and the Hamiltonian in Eq. (7) reduces to $H^{\text{ZQ}}=d_{\text{IS}}I_{x}^{\text{ZQ}}$ ( $d_{\text{IS}}$ is time independent). The spin state represented by the operator $I_{z}^{\text{ZQ}}$ is rotated around the $I_{x}^{\text{ZQ}}$ axis as a consequence of the dipolar interaction. Simultaneously, the spin state $I_{z}^{\text{DQ}}$ evolves in the DQ subspace. We can assume that $\omega_{I}+\omega_{S}$ is much larger than $d_{\text{IS}}$ . The effective rotation axis is thus oriented along $I_{z}^{\text{DQ}}$ ; see Eq. (8). As a result, $H^{\text{DQ}}$ has no effect on the $I_{z}^{\text{DQ}}$ state. This is summarized in the following equations.

9IzZQ⟶HZQIzZQcos(dISt)-IyZQsin(dISt)=12(Iz-Sz)cos(dISt)-(IySx-IxSy)sin(dISt)10IzDQ⟶HDQIzDQ=12Iz+Sz11Iz=IzZQ+IzDQ⟶HZQ+HDQIz12[cos(dISt)+1]+Sz12[1-cos(dISt)]-(IySx-IxSy)sin(dISt)

The $I_{z}$ spin state is transformed into $S_{z}$ when $\text{cos}(d_{\text{IS}}t)=-1$ , resulting in a full inversion of the $I_{z}^{\text{ZQ}}$ operator.

For the double-quantum Hartmann–Hahn condition $\omega_{I}+\omega_{S}=0$ , the rotation occurs in the DQ subspace. By analogy with the previous case, we assume $\left|\omega_{I}-\omega_{S}\right|\gg d_{\text{IS}}$ . Under this precondition, the ZQ spin state is not changed.

12IzZQ⟶HZQIzZQ=12(Iz-Sz)13IzDQ⟶HDQIzDQcos(dISt)-IyDQsin(dISt)=12(Iz+Sz)cos(dISt)-(IySx+IxSy)sin(dISt)14Iz=IzZQ+IzDQ⟶HZQ+HDQIz12[cos(dISt)+1]-Sz12[1-cos(dISt)]-(IySx+IxSy)sin(dISt)

For $\text{cos}(d_{\text{IS}}t)=-1$ , the $I_{z}^{\text{DQ}}$ operator is inverted resulting in the generation of the operator $-S_{z}$ . Note that the double-quantum Hartmann–Hahn condition yields negative signal intensity.

The dipolar coupling is an orientation-dependent interaction. To yield the magnetization transfer dynamics for a powder sample, the ensemble of all possible crystallite orientations has to be accounted for. The powder-averaged inversion efficiency is lower since the condition of a complete transfer, $\text{cos}(d_{\text{IS}}t)=-1$ , will hold only for a single orientation.

### 2.3 Magic angle spinning and average Hamiltonians

In the case of MAS, the Hamiltonians become time dependent. The analysis is then performed using average Hamiltonian theory (AHT) employing the Magnus expansion. A tutorial on AHT principles was presented by Brinkmann (2016). To retain fast convergence of the Magnus series, the Hamiltonian is expressed in an appropriate interaction frame. Equation (2) implies four resonance conditions upon transformation into a new rotating frame in which the periodic modulations of $d_{\text{IS}}(t)$ are removed by application of RF fields. These resonance conditions are associated with the characteristic frequencies $n\omega_{R}$ with n=±1,±2 . We choose n=+1 and focus on the ZQ subspace. In general, transformation to a new reference frame is described using a propagator $U_{T}(t)$ . This propagator transforms the Hamiltonian according to

15 $$H^{\prime }=U_{T}^{+}(t)HU_{T}(t)-iU_{T}^{+}(t)\frac{d}{dt}U_{T}(t).$$

In this case, $U_{T}(t)=\text{exp}(-i\omega_{R}tI_{z}^{\text{ZQ}})$ . The transformation can be regarded as a rotation around $I_{z}^{\text{ZQ}}$ with a frequency $-\omega_{R}$ . The second term in Eq. ([Disp-formula Ch1.E15]) is a Coriolis term which introduces the term $-\omega_{R}I_{z}^{\text{ZQ}}$ into the transformed Hamiltonian.

16 $$\begin{array}{rl} H^{{\text{ZQ}}^{\prime }}= & \;(\omega_{I}-\omega_{S}-\omega_{R})I_{z}^{\text{ZQ}}\\ & +d_{\text{IS}}(t)\left(I_{x}^{\text{ZQ}}\text{cos}(\omega_{R}t)-I_{y}^{\text{ZQ}}\text{sin}(\omega_{R}t)\right) \end{array}$$

The first-order Hamiltonian is the time average over the modulation period $\tau_{R}=2\pi /\omega_{R}$

17 $${\bar{H}}^{\text{ZQ}}=\frac{1}{\tau_{R}}\int_{0}^{\tau_{R}}H^{{\text{ZQ}}^{\prime }}dt.$$

The integral over the time-dependent parts in Eq. ([Disp-formula Ch1.E16]) is evaluated as follows (making use of trigonometric identities):

$$\begin{array}{rl} & \frac{1}{\tau_{R}}\int_{0}^{\tau_{R}}[g_{1}\text{cos}(\omega_{R}t+\gamma )+g_{2}\text{cos}(2\omega_{R}t+2\gamma )]\\ & \times \left(I_{x}^{\text{ZQ}}\text{cos}(\omega_{R}t)-I_{y}^{\text{ZQ}}\text{sin}(\omega_{R}t)\right)dt=\\ & =\frac{1}{\tau_{R}}\int_{0}^{\tau_{r}}\{g_{1}\frac{1}{2}[\text{cos}(2\omega_{R}t+\gamma )+\text{cos}(\gamma )]\\ & +g_{2}\frac{1}{2}[\text{cos}(3\omega_{R}t+2\gamma )+\text{cos}(\omega_{R}t+2\gamma )]\}dtI_{x}^{\text{ZQ}}+\\ & -\frac{1}{\tau_{R}}\int_{0}^{\tau_{r}}\{g_{1}\frac{1}{2}[\text{sin}(2\omega_{R}t+\gamma )-\text{sin}(\gamma )]\\ & +g_{2}\frac{1}{2}[\text{sin}(3\omega_{R}t+2\gamma )-\text{sin}(\omega_{R}t+2\gamma )]\}dtI_{y}^{\text{ZQ}}=\\ & =\frac{1}{2}g_{1}\text{cos}(\gamma )I_{x}^{\text{ZQ}}+\frac{1}{2}g_{1}\text{sin}(\gamma )I_{y}^{\text{ZQ}}. \end{array}$$

We thus obtain the first-order average Hamiltonian in the ZQ subspace as

18 $$\begin{array}{rl} {\bar{H}}^{\text{ZQ}}= & \;(\omega_{I}-\omega_{S}-\omega_{R})I_{z}^{\text{ZQ}}\\ & +\frac{1}{2}g_{1}\left(\text{cos}\gamma I_{x}^{\text{ZQ}}+\text{sin}\gamma I_{y}^{\text{ZQ}}\right). \end{array}$$

The Hartmann–Hahn condition is corrected to account for the rotation of the sample and has the form $\omega_{I}-\omega_{S}=\omega_{R}$ . In this case, the component of ${\bar{H}}^{\text{ZQ}}$ along the $I_{z}^{\text{ZQ}}$ axis vanishes and the dipolar interaction results in a rotation around an axis in the transversal plane, with a phase depending on γ . For each crystallite, the spin state $I_{z}^{\text{ZQ}}$ is flipped away from the z axis generating a transversal component. These transversal components are equally distributed with respect to the γ angle and average to 0 in a powder sample. Only the projection on the $I_{z}^{\text{ZQ}}$ axis is relevant, and we can therefore arbitrarily set γ=0 .

The calculation can be repeated for other choices of n and the following zero-quantum average Hamiltonians are obtained:

19 $${\bar{H}}^{\text{ZQ}}=\left(\omega_{I}-\omega_{S}-n\omega_{R}\right)I_{z}^{\text{ZQ}}+\frac{1}{2}g_{n}I_{x}^{\text{ZQ}}.$$

The fast convergence of the Magnus expansion is maintained and the proper description of spin dynamics by an average Hamiltonian is valid in the vicinity of the Hartmann–Hahn condition ( $\omega_{I}-\omega_{S}-n\omega_{R}=0)$ . The RF amplitudes $\omega_{I}$ and $\omega_{S}$ may become time dependent if a linear ramp or an adiabatic sweep is applied. In any case, we assume that RF changes are slow compared to the MAS frequency to ensure the validity of this treatment.

The analysis is completed by inspecting the spin dynamics in the DQ subspace. We apply the same procedure as for the ZQ subspace, yielding

20 $${\bar{H}}^{\text{DQ}}=(\omega_{I}+\omega_{S}-n\omega_{R})I_{z}^{\text{DQ}}+\frac{1}{2}g_{n}I_{x}^{\text{DQ}}.$$

For the zero-quantum condition, it is assumed that the $I_{Z}^{\text{DQ}}$ term dominates the average Hamiltonian ${\bar{H}}^{\text{DQ}}$ ; i.e., $\omega_{I}+\omega_{S}-n\omega_{R}\gg d_{\text{IS}}$ for all n=±1,±2 . Under these conditions, the initial state $I_{z}^{\text{DQ}}$ remains unchanged. However, these conditions might be violated for large RF amplitude sweeps or in the case of substantial RF field inhomogeneity.

### 2.4 CP matching profiles

For constant RF amplitudes, the magnetization transfer process can be analytically described to derive the so-called CP matching profiles (sometimes dubbed Hartmann–Hahn fingers). This derivation was previously published in Levitt (1991) and Wu and Zilm (1993). It is assumed that both the ZQ and DQ Hartmann–Hahn conditions are independent. We reiterate the calculation for the matching condition and focus first on the ZQ Hamiltonian given in Eq. ([Disp-formula Ch1.E19]). We proceed with the final transformation into the effective field of the Hamiltonian. The Hamiltonian ${\bar{H}}^{\text{ZQ}}$ can be represented as a vector in the xz plane. This vector has an angle ϕ with the x axis. The transformation into the effective field is described by a rotation around $I_{y}^{\text{ZQ}}$ by an angle -ϕ , which is equivalent to the application of the propagator $U_{T}=\text{exp}(-i\phi I_{y}^{\text{ZQ}})$ . It makes the x axis of the new frame coincide with the effective Hamiltonian vector. Note that the Coriolis term in Eq. ([Disp-formula Ch1.E15]) is absent because $U_{T}$ is time independent. The effective Hamiltonian can be written as

21H‾effZQ=ωeffZQ,nIxeff,22ωeffZQ,n=(ωI-ωS-nωR)2+14gn2,23tanϕ=ωI-ωS-nωR12gn.

The initial spin state $\rho^{\text{ZQ}}(0)=I_{z}^{\text{ZQ}}$ transforms into $\rho^{\text{eff}}(0)=U_{T}^{+}\rho^{\text{ZQ}}(0)U_{T}=\text{cos}\phi I_{z}^{\text{eff}}+\text{sin}\phi I_{x}^{\text{eff}}$ in the effective field frame and evolves with a frequency $\omega_{\text{eff}}^{\text{ZQ},n}$ around the effective field axis $I_{x}^{\text{eff}}$ :

24 $$\begin{array}{rl} \rho^{\text{eff}}(t) & =\text{cos}\phi \left(I_{z}^{\text{eff}}\text{cos}(\omega_{\text{eff}}^{\text{ZQ},n}t)-I_{y}^{\text{eff}}\text{sin}(\omega_{\text{eff}}^{\text{ZQ},n}t)\right)+\text{sin}\phi I_{x}^{\text{eff}}\\ & =\text{sin}\phi I_{x}^{\text{eff}}-\text{cos}\phi \text{sin}(\omega_{\text{eff}}^{\text{ZQ},n}t)I_{y}^{\text{eff}}+\text{cos}\phi \text{cos}(\omega_{\text{eff}}^{\text{ZQ},n}t)I_{z}^{\text{eff}}. \end{array}$$

The result is transformed back from the effective field frame into the ZQ subspace as $\rho^{\text{ZQ}}(t)=U_{T}\rho^{\text{eff}}(t)U_{T}^{+}$ . This yields

25 $$\begin{array}{rl} \rho^{\text{ZQ}}(t)= & \;\text{sin}\phi \left(I_{x}^{\text{ZQ}}\text{cos}\phi +I_{z}^{\text{ZQ}}\text{sin}\phi \right)-\text{cos}\phi \text{sin}(\omega_{\text{eff}}^{\text{ZQ},n}t)I_{y}^{\text{ZQ}}\\ & +\text{cos}\phi \text{cos}(\omega_{\text{eff}}^{\text{ZQ},n}t)\left(I_{z}^{\text{ZQ}}\text{cos}\phi -I_{x}^{\text{ZQ}}\text{sin}\phi \right)\\ = & \;\text{sin}\phi \text{cos}\phi \left[1-\text{cos}(\omega_{\text{eff}}^{\text{ZQ},n}t)\right]I_{x}^{\text{ZQ}}-\text{cos}\phi \text{sin}(\omega_{\text{eff}}^{\text{ZQ},n}t)I_{y}^{\text{ZQ}}\\ & +\left[{\text{sin}}^{2}\phi +{\mathrm{cos}}^{2}\phi \text{cos}(\omega_{\text{eff}}^{\text{ZQ},n}t)\right]I_{z}^{\text{ZQ}}. \end{array}$$

Equation (25) describes the trajectory of the $I_{z}^{\text{ZQ}}$ operator in the ZQ subspace under the influence of the RF pulses applied in the CP experiment. For evaluation of the magnetization transfer process, only the projection on the $I_{z}^{\text{ZQ}}$ axis is important. We assume that there is no evolution in the DQ subspace; i.e., $\rho^{\text{DQ}}(t)=I_{z}^{\text{DQ}}$ . The initial $I_{z}$ operator thus evolves as (recall $I_{z}=I_{z}^{\text{ZQ}}+I_{z}^{\text{DQ}}$ )

26 $$\begin{array}{rl} \rho^{\text{ZQ}}(t)+\rho^{\text{DQ}}(t)= & \;\left({\text{sin}}^{2}\phi +{\mathrm{cos}}^{2}\phi \text{cos}(\omega_{\text{eff}}^{\text{ZQ},n}t)\right)\\ & \times \frac{1}{2}(I_{z}-S_{z})+\frac{1}{2}(I_{z}+S_{z}). \end{array}$$

We obtain the CP transfer efficiency in the vicinity of the zero-quantum condition ( n ) by collecting the terms in front of the $S_{z}$ operator:

ϵZQ,n=121-sin2ϕ-cos⁡2ϕcos(ωeffZQ,nt)=12cos⁡2ϕ-cos⁡2ϕcos(ωeffZQ,nt)=cos⁡2ϕ21-cos(ωeffZQ,nt)27ϵZQ,n=1214gn2(ωI-ωS-nωR)2+14gn21-cos(ωeffZQ,nt).

A similar calculation for the double-quantum Hartmann–Hahn condition yields

28ϵDQ,n=-1214gn2(ωI+ωS-nωR)2+14gn21-cos(ωeffDQ,nt),29ωeffDQ,n=(ωI+ωS-nωR)2+14gn2.

Note the negative sign of the transferred magnetization for the double-quantum Hartmann–Hahn transfer. Equations (27) and (28) are identical to the result of an alternative derivation presented by Marica and Snider (2003). The CP MAS matching profile has the form of a Lorentzian function with a width that is dependent on the dipolar coupling $b_{\text{IS}}$ and the crystallite orientation (Euler angle β) that are included in the $g_{n}$ factors. In powders, a quantitative magnetization transfer is not possible as a consequence of the dependence of the size of the effective dipolar coupling on orientation. The magnetization transfer efficiency under MAS is independent of the γ angle. This property is referred to as γ encoding. The powder average is obtained by evaluation of the integral:

30 $$\langle \epsilon^{\text{ZQ},n}\rangle_{\text{powder}}=\frac{1}{2}\int_{0}^{\pi }\epsilon^{\text{ZQ},n}\text{sin}\beta d\beta .$$

**Figure 1:**
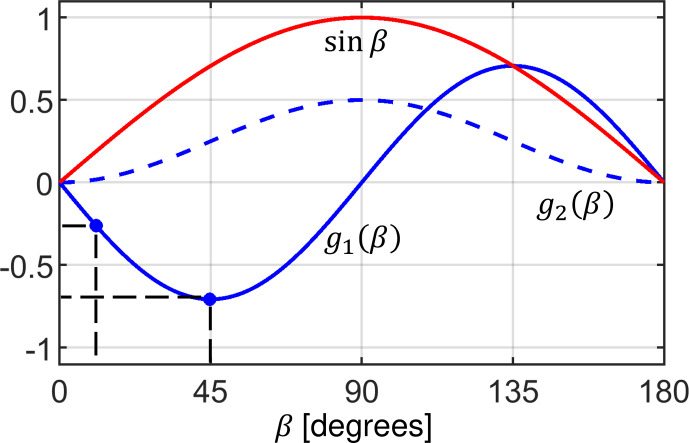
Dipolar coupling scaling factors $g_{1}(\beta )$ (solid blue line) and $g_{2}(\beta )$ (dashed blue line) defined in Eqs. (3) and (4). The red curve represents the relative probability of finding a specific orientation in a powder sample. This weighting factor is employed for the calculation of the transfer efficiencies ϵ in Eq. ([Disp-formula Ch1.E30]). β angles with β = 15 and 45 ${}^{\circ }$ are used for the visualization of the spin dynamics in the “Results and discussion” section.

### 2.5 Radiofrequency field inhomogeneity

**Figure 2:**
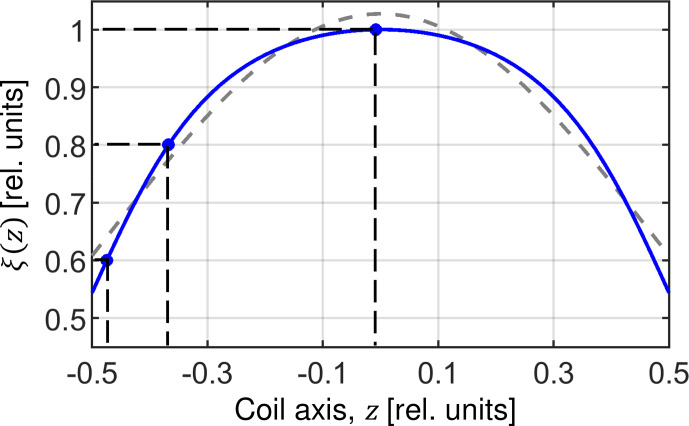
RF field inhomogeneity profile along the axis of a solenoid coil. The profile is calculated according to Engelke (2002) assuming a coil length of 7.9 mm , a diameter of 3.95 mm and seven turns (blue line). The dashed gray line represents a fit of the RF profile assuming a Gaussian function suggested by Paulson et al. (2004). The power law relation introduced by Gupta et al. (2015) yields a perfect fit of the theoretical behavior and exactly matches the blue curve. Values ξ = 0.6, 0.8 and 1.0 are used in the “Results and discussion” section to visualize spin dynamics.

Radiofrequency fields in MAS probes are realized using solenoid coils. However, a solenoid produces a rather inhomogeneous distribution of magnetic fields across the sample (Tošner et al., 2017). Moreover, as the sample rotates, individual spin packets travel along circles through a spatially inhomogeneous RF field which is determined by the helical geometry of the solenoid coil. This RF inhomogeneity introduces periodic modulations of both the RF amplitude and phase. For the special case of the CP experiment, it was recently shown that these temporal modulations have a negligible effect (Aebischer et al., 2021) and will be ignored in the present treatment. In addition, the distribution of the RF fields depends on the frequency (Engelke, 2002) and can be influenced by different balancing of the RF circuitry on different channels (Paulson et al., 2004). For simplicity, we assume the RF field distributions to be equal for the I and S spins and disregard the radial dependency. The effect of RF field inhomogeneity on the CP experiment was previously studied by Paulson et al. (2004) and Gupta et al. (2015). An example of the distribution of the RF field along the coil axis, denoted ξ(z) , is shown in Fig. 2. As noted by Gupta et al. (2015), the profile deviates from a Gaussian function and is described well by a power law dependence. In our study, we use the $B_{1}$ profile calculated according to Engelke (2002).

The distribution of RF field amplitudes enters the formulas of the CP experiment using the substitution

31 $$\begin{array}{rl} & \omega_{I}\overset{\text{replace}}{⟶}\xi (z)\omega_{I}^{\text{NOM}},\\ & \omega_{S}\overset{\text{replace}}{⟶}\xi (z)\omega_{S}^{\text{NOM}}, \end{array}$$

where $\omega_{I}^{\text{NOM}}$ and $\omega_{S}^{\text{NOM}}$ refer to the nominal RF amplitudes realized in the center of the coil ( z=0 where ξ(0)=1) . The overall experimental efficiency corresponds to the integral over the sample volume weighted by the detection sensitivity of the coil. According to the reciprocity theorem (Hoult, 2000), the sensitivity is proportional to the RF field. We assume that the sample extends over a length l and is placed symmetrically within the solenoid coil.

32 $$\langle \epsilon^{\text{ZQ},n}\rangle_{\text{powder}}^{\text{rf-inh}}=\frac{1}{w}\int_{-l/2}^{+l/2}\langle \epsilon^{\text{ZQ},n}\rangle_{\text{powder}}\xi (z)dz$$

The normalization factor w is given as

33 $$w=\int_{-l/2}^{+l/2}\xi (z)dz.$$

It is not possible to match the Hartmann–Hahn conditions for the whole sample volume. Assuming that the zero-quantum condition is fulfilled for the nominal RF amplitudes, i.e., $\omega_{I}^{\text{NOM}}-\omega_{S}^{\text{NOM}}=n\omega_{R}$ , we get

$$\begin{array}{rl} \omega_{I}-\omega_{S}-n\omega_{R} & =\xi (z)\left(\omega_{I}^{\text{NOM}}-\omega_{S}^{\text{NOM}}\right)-n\omega_{R}\\ & =\xi (z)n\omega_{R}-n\omega_{R}=n\omega_{R}[\xi (z)-1] \end{array}$$

and

34 $${\bar{H}}^{\text{ZQ}}=n\omega_{R}[\xi (z)-1]I_{z}^{\text{ZQ}}+\frac{1}{2}g_{n}I_{x}^{\text{ZQ}}.$$

Equation ([Disp-formula Ch1.E34]) shows that in the case of an inhomogeneous RF field, the prevailing component along the $I_{z}^{\text{ZQ}}$ operator in the effective Hamiltonian ${\bar{H}}^{\text{ZQ}}$ is proportional to the MAS frequency $\omega_{R}$ , multiplied by the order of the recoupling condition n . The effect of RF amplitude mismatch on spin dynamics is more pronounced for small dipolar couplings, $b_{\text{IS}}$ , which is reflected in the width of the CP MAS matching profiles derived above. Thus, we could analytically derive a dependence of the performance of the CP experiment on the MAS frequency.

### 2.6 Linear ramp and adiabatic sweep

**Figure 3:**
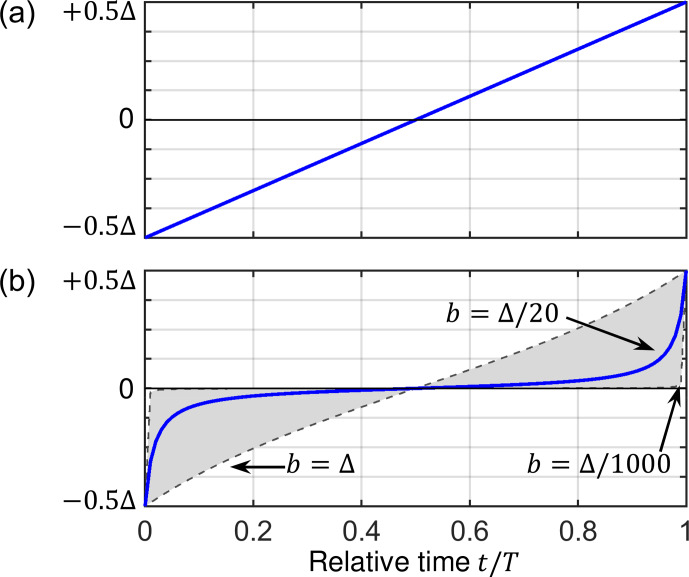
RF amplitude sweeps employed in cross-polarization experiments for **(a)** a linear ramp and **(b)** an adiabatic tangential sweep. Equations ([Disp-formula Ch1.E36]) and ([Disp-formula Ch1.E37]) mathematically describe the time-dependent RF amplitude. The parameter b determines the curvature of the adiabatic tangential shape.

The most popular way to overcome the limitations of the constant amplitude CP and the RF mismatch at different positions of the sample is the use of a linear ramp or an adiabatic tangential sweep on one of the RF channels. We can define

35 $$\omega_{I}^{\text{NOM}}=\omega_{I}^{0}+f(t),$$

where the function f(t) describes the sweep from -Δ/2 to +Δ/2 over time t∈〈0,T〉 . The function f(t) can be defined for the linear ramp as

36 $$f(t)=\Delta \left(\frac{t}{T}-\frac{1}{2}\right)$$

and for a tangential sweep as

37 $$f(t)=b\text{tan}\left[\left(\frac{2t}{T}-1\right)\mathrm{arctan}\frac{\Delta }{2b}\right],$$

where b parameterizes the curvature of the sweep. Values for b are typically in the range of $\frac{\Delta }{1000}<b<\Delta$ . For $b=\frac{\Delta }{1000}$ , f(t) is almost constant except for the end points where the function changes rapidly. For b=Δ , f(t) approaches the linear ramp. The influence of b on the shape is illustrated in Fig. 3. During a truly adiabatic transfer, the effective field is aligned with the initial magnetization along the $+I_{z}^{\text{ZQ}}$ axis and changes its orientation slowly towards $-I_{z}^{\text{ZQ}}$ . The spin state is locked along the effective field and is inverted as well (Hediger et al., 1995). The adiabaticity condition is given as

38 $$\frac{d}{dt}\phi (t)\ll \omega_{\text{eff}},$$

where $\omega_{\text{eff}}$ is defined in Eq. (22) and the angle ϕ is given in Eq. (23). Adiabatic inversion pulses have been an integral part of the NMR toolbox for a long time (Baum et al., 1985). There is, however, a substantial difference between broadband inversion pulses and cross-polarization. Inversion pulses allow us to manipulate the effective field along z and x directions, corresponding to offset and RF amplitude, respectively. In the CP experiment, the x axis component of the effective Hamiltonian is fixed and is determined by the dipolar coupling; see Eqs. ([Disp-formula Ch1.E19]) and ([Disp-formula Ch1.E20]). In addition, perfect alignment of the effective field with the initial state is difficult to achieve as the RF amplitudes are restricted to the vicinity of the Hartmann–Hahn condition.

### 2.7 RF amplitude sweeps and RF field inhomogeneity

In the following, we aim to include RF field inhomogeneity in the description of the RF amplitude sweep of Eq. ([Disp-formula Ch1.E35]). We assume that the zero-quantum Hartmann–Hahn matching conditions are fulfilled in the middle of the sweep and in the center of the coil for the nominal RF field amplitudes, i.e., for $\omega_{I}^{0}-\omega_{S}^{\text{NOM}}=n\omega_{R}$ . The $I_{z}^{\text{ZQ}}$ component of the Hamiltonian ${\bar{H}}^{\text{ZQ}}$ then becomes

39 $$\begin{array}{rl} \omega_{I}-\omega_{S}-n\omega_{R} & =\xi (z)\left[\omega_{I}^{\text{NOM}}-\omega_{S}^{\text{NOM}}\right]-n\omega_{R}\\ & =\xi (z)\left[\omega_{I}^{0}+f(t)-\omega_{S}^{\text{NOM}}\right]-n\omega_{R}\\ & =\xi (z)[f(t)+n\omega_{R}]-n\omega_{R}\\ & =\xi (z)f(t)+n\omega_{R}[\xi (z)-1] \end{array}$$

and

40 $${\bar{H}}^{\text{ZQ}}=\left\{\xi (z)f(t)+n\omega_{R}[\xi (z)-1]\right\}I_{z}^{\text{ZQ}}+\frac{1}{2}g_{n}I_{x}^{\text{ZQ}}.$$

**Figure 4:**
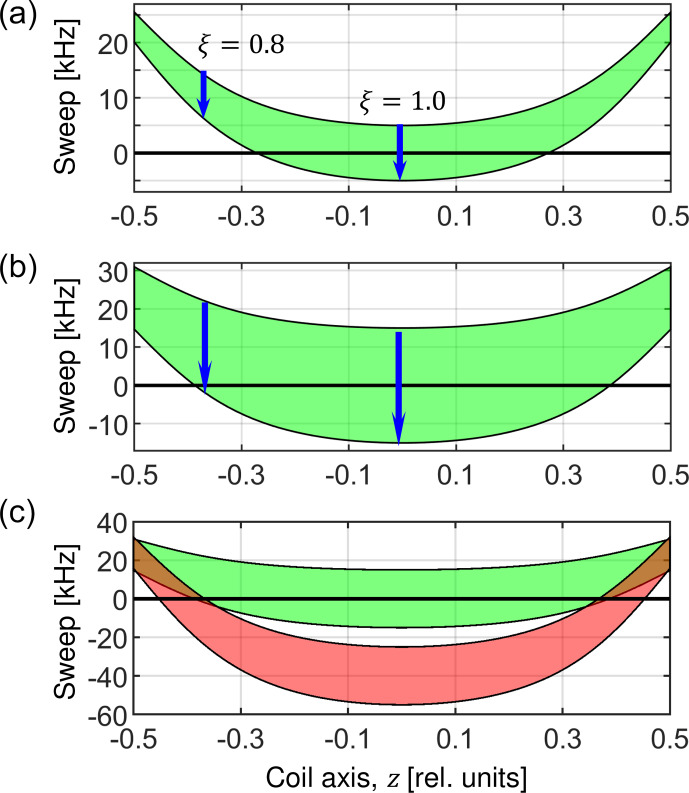
Visualization of the RF sweep ranges as a function of the position of a particular spin packet along the coil axis. The Hartmann–Hahn resonance condition is artificially defined for a sweep frequency 0 kHz . **(a)** The sweep range (green area) is evaluated according to Eq. ([Disp-formula Ch1.E39]) for n=+1 and assuming a MAS frequency of 50 kHz . The blue arrows indicate the direction of the sweep for an RF inhomogeneity factor of ξ = 0.8 and 1.0. The sweep amplitude Δ corresponds to 10 and 30 kHz in **(a)** and **(b)**, respectively. **(c)** Overlay of the RF amplitude sweeps evaluated for the ZQ ( n=+1 ) matching condition (Eq. [Disp-formula Ch1.E39], green) and DQ ( n=+2 ) matching condition (Eq. [Disp-formula Ch1.E41], red) with nominal RF amplitudes $\omega_{I}^{\text{NOM}}/2\pi$ = 95 kHz and $\omega_{S}^{\text{NOM}}/2\pi$ = 45 kHz . These values were selected to demonstrate that the ZQ matching condition is satisfied in the center of the coil, and simultaneously a DQ is encountered for spin packets in regions of the sample where the RF amplitudes are scaled down by the RF field inhomogeneity.

Now, the sweep function f(t) is scaled by the RF field inhomogeneity factor ξ(z) . At the same time, the center of the sweep is shifted by an amount proportional to the MAS frequency $\omega_{R}$ . In Fig. 4, the sweep range is depicted in green as a function of position along the coil axis. Spins located in volume elements towards the ends of the coil where the RF field is smaller experience RF amplitude sweeps that do not cover the recoupling condition at all (e.g., for ξ = 0.8 in Fig. 4a). This is another example of how increased MAS frequencies impact the cross-polarization experiment and cause a decrease in performance.

When setting the numerical values of RF amplitudes $\omega_{I}^{0}$ , $\omega_{S}^{\text{NOM}}$ and the sweep range Δ , it can happen that double-quantum conditions are fulfilled in some places within the sample when the values are scaled by the RF field inhomogeneity. The double-quantum conditions are governed by the formula

41 $$\begin{array}{rl} \omega_{I}+\omega_{S}-n\omega_{R} & =\xi (z)\left[\omega_{I}^{\text{NOM}}+\omega_{S}^{\text{NOM}}\right]-n\omega_{R}\\ & =\xi (z)\left[\omega_{I}^{0}+f(t)+\omega_{S}^{\text{NOM}}\right]-n\omega_{R}\\ & =\xi (z)f(t)+\xi (z)\left(\omega_{I}^{0}+\omega_{S}^{\text{NOM}}\right)-n\omega_{R}, \end{array}$$

which is represented in red in Fig. 4c. While the values $\omega_{I}^{0}$ and $\omega_{S}^{\text{NOM}}$ do satisfy the zero-quantum n=+1 condition around the center of the coil, at the same time, they satisfy the double-quantum n=+2 condition towards the ends of the coil (places where the red area crosses the zero value). As a result, there are parts of the sample that produce a positive magnetization transfer and parts that experience a negative transfer. Thus, the overall efficiency of the experiment is decreased.

## 3 Results and discussion

### 3.1 CP matching profile

**Figure 5:**
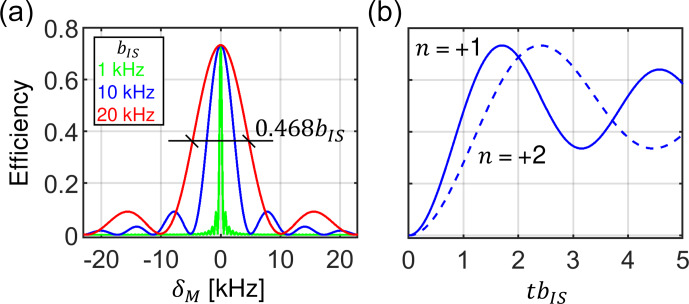
Properties of the constant amplitude CP experiment assuming homogeneous RF fields. **(a)** The width of the CP matching profile around the zero-quantum ( n=+1 ) Hartmann–Hahn matching condition depends on the dipolar coupling strength $b_{\text{IS}}$ . **(b)** Magnetization buildup of the transferred magnetization for the n=+1 and n=+2 matching condition. Independently of the MAS frequency and $b_{\text{IS}}$ , the n=+2 condition reaches the same maximum, however, at longer mixing times. The curves were calculated using Eqs. (27) and ([Disp-formula Ch1.E30]).

Experimentally, optimal cross-polarization conditions are found in experiments in which the RF amplitude on one of the RF channels is systematically varied to yield the highest sensitivity. If the Hartmann–Hahn recoupling condition is very narrow, this can be difficult as many repetitions with a small increment in the RF amplitude are required. In the Theory section, we derived analytical formulas for the CP matching profiles for constant RF amplitudes. We have found that for a homogeneous RF field distribution, the width at half height of the recoupling condition is governed by the size of the dipolar coupling and can be estimated as $0.468b_{\text{IS}}$ after powder averaging. Both the width and the maximal transfer efficiency are independent of the MAS frequency. A maximum transfer of 73 % is achieved for mixing times satisfying the condition $tb_{\text{IS}}=1.7$ for the n = ± 1 recoupling conditions. The same efficiency is obtained for the n = ± 2 conditions. However, due to the different spatial dependence and scaling factors in $g_{1}$ and $g_{2}$ terms (Eqs. 3 and 4), the maximum is achieved there for mixing times $tb_{\text{IS}}=2.4$ . These facts are well known and are presented graphically in Fig. 5. Figure 5a shows the CP matching profile calculated using Eqs. (27) and ([Disp-formula Ch1.E30]) for n=+1 and assuming a dipolar coupling constant $b_{\text{IS}}$ of 1, 10 and 20 kHz , which are the characteristic values for ${}^{13}C$ – ${}^{15}N$ , ${}^{1}H$ – ${}^{15}N$ and ${}^{1}H$ – ${}^{13}C$ spin pairs, respectively. $\langle \epsilon^{\text{ZQ},1}\rangle_{\text{powder}}$ is represented as a function of the RF amplitude mismatch $\delta_{M}/2\pi =\omega_{I}-\omega_{S}-\omega_{R}$ with respect to the exact Hartmann–Hahn.

**Figure 6:**
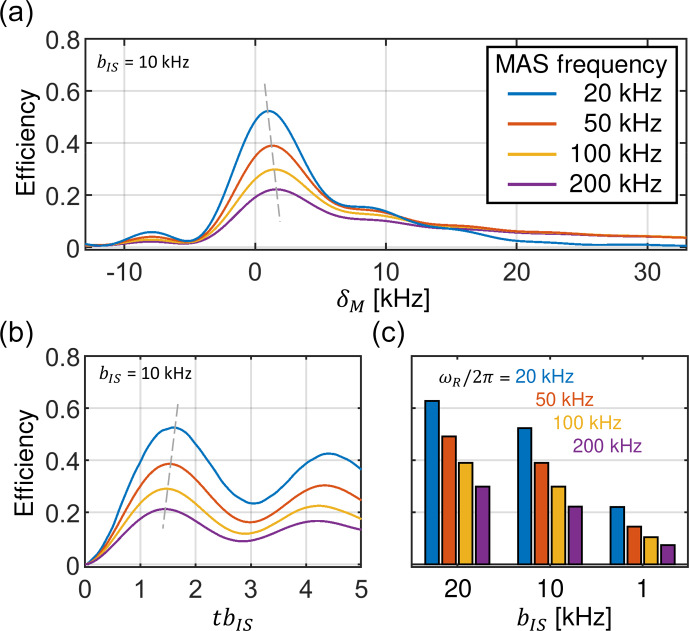
Transfer efficiency of the constant amplitude CP experiment in the presence of RF field inhomogeneity and assuming a dipolar coupling strength $b_{\text{IS}}$ = 10 kHz . For the calculation, a rotor fully packed with material is assumed. **(a)** The maximum of the CP matching profile decreases with increasing MAS frequency for the zero-quantum ( n=+1 ) condition. At the same time, the width increases. A dashed gray line is used to indicate the position of the maximum. The maximum of the CP matching profile shifts to higher mismatch values $\delta_{M}$ for increased MAS frequencies. **(b)** Magnetization buildup curves for different MAS frequencies. The legend is indicated in panel **(a)**. With increasing MAS frequencies, magnetization reaches the maximum transfer at shorter mixing times. **(c)** Maximum transfer efficiencies for the characteristic dipolar coupling values $b_{\text{IS}}$ of 1, 10 and 20 kHz for different MAS frequencies. Data were generated using Eqs. (27), (31) and ([Disp-formula Ch1.E32]).

For inhomogeneous RF fields, the CP matching profile can be quantitatively described by inserting Eq. (31) into Eq. (27) and taking the average in Eq. ([Disp-formula Ch1.E32]). Figure 6a shows the influence of inhomogeneous RF fields and the induced asymmetric broadening of the matching profile $\langle \epsilon^{\text{ZQ},+1}\rangle_{\text{powder}}^{\text{rf-inh}}$ . Clearly, the maximal transfer efficiency substantially decreases with increasing MAS frequency.

A closer inspection of the CP matching profiles in Fig. 6a reveals that the maximum overall transfer efficiency is not reached for the exact ZQ ( n=+1 ) condition with $\omega_{I}^{\text{NOM}}-\omega_{S}^{\text{NOM}}=\omega_{R}$ , corresponding to $\delta_{M}$ = 0. In practice, it is advantageous to set $\omega_{I}^{\text{NOM}}$ a little higher and thus shift the volume element where the Hartmann–Hahn condition is matched away from the center of the coil. This allows us to partially compensate for the destructive effect of the RF field inhomogeneity. This mismatch $\delta_{M}$ of the Hartmann–Hahn matching condition is naturally found during the experimental setup when the RF fields are optimized to experimentally yield the best efficiency. However, the mismatch is small (a few kHz at most) and generally decreases with decreasing MAS frequency (see the dashed line in Fig. 6a). Similarly, the RF field inhomogeneity has a subtle effect on the buildup of the transferred magnetization. Figure 6b shows that maximum transfer occurs at shorter mixing times for increased MAS frequencies.

Figure 6c shows how decreasing dipolar couplings result in a diminished Hartmann–Hahn transfer efficiency. The calculations are carried out for three typical dipolar coupling values and for MAS frequencies in the range of 20 to 200 kHz . Strikingly, for $\omega_{R}/2\pi$ = 200 kHz and $b_{\text{IS}}$ = 1 kHz , the maximum transfer is only about 7 %.

We used numerical simulations in SIMPSON (Bak et al., 2000; Tošner et al., 2014) to verify the predictions of the analytical model. To implement an experiment, specific values of $\omega_{I}$ and $\omega_{S}$ need to be selected. A consideration of RF field inhomogeneity increases the complexity of this selection process, since certain values of $\omega_{I}$ and $\omega_{S}$ can lead to a situation in which ZQ and DQ recoupling conditions are fulfilled simultaneously in different parts of the sample (Fig. 4c). This phenomenon was explored experimentally by Gupta et al. (2015). If this situation is avoided, we find perfect agreement between the analytical model and the numerical simulations.

**Figure 7:**
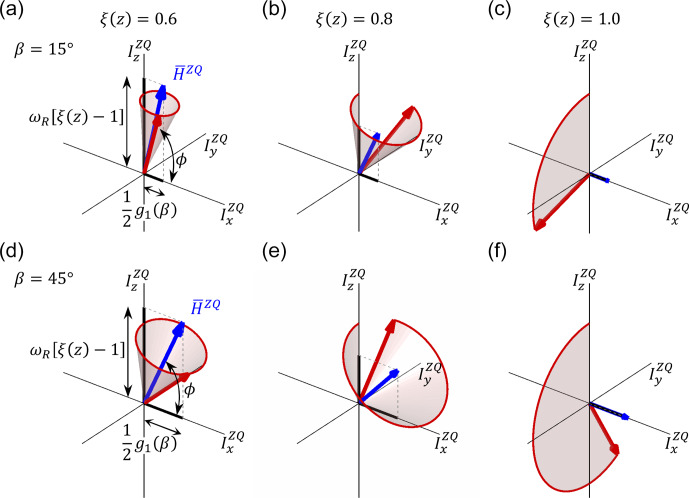
Visualization of the spin state trajectories for the constant amplitude cross-polarization experiment evaluated for two crystal orientations. **(a–c)** Crystallite orientation β = 15 ${}^{\circ }$ ; **(d–f)** β = 45 ${}^{\circ }$ . The calculations were carried out for three positions along the coil axis that correspond to RF field scaling values of ξ(z) = 0.6 (panels **a** and **d**), 0.8 (panels **b** and **e**) and 1.0 (panels **c** and **f**). The state vector, $\rho^{\text{ZQ}}$ , is represented by a red vector. The effective Hamiltonians are represented by blue vectors. $\rho^{\text{ZQ}}$ rotates around ${\bar{H}}^{\text{ZQ}}$ on the surface of a cone (shaded area). In the simulation, a MAS frequency of 50 kHz and $b_{\text{IS}}$ = 10 kHz was assumed.

### 3.2 Visualization of the magnetization transfer trajectories

In the following, we aim to visualize the spin trajectory during the CP experiment in its basic form with constant RF and with RF amplitude sweeps. We focus on the vicinity of the ZQ ( n=+1 ) Hartmann–Hahn condition and use the effective Hamiltonian ${\bar{H}}^{\text{ZQ}}$ given in Eq. ([Disp-formula Ch1.E34]) for the analysis. We consider RF field inhomogeneity and assume nominal RF amplitudes that match the recoupling condition in the center of the coil: $\omega_{I}^{\text{NOM}}-\omega_{S}^{\text{NOM}}=\omega_{R}$ . Figure 7 shows the spin dynamics for two crystallite orientations ( β = 15 and 45 ${}^{\circ }$ ) and three positions within the coil ( ξ = 0.6, 0.8 and 1.0). These conditions are highlighted in Figs. 1 and 2. In the center of the coil where ξ = 1.0, the Hamiltonian ${\bar{H}}^{\text{ZQ}}$ (blue vector) is aligned with the $I_{x}^{\text{ZQ}}$ axis. The spin state vector $\rho^{\text{ZQ}}(t)$ (red vector) rotates in circles within the yz plane with an angular velocity that depends on the crystallite orientation (Fig. 7c and f). This situation corresponds to the case without RF field inhomogeneity.

Depending on the position within the coil, a mismatch contribution in the effective Hamiltonian ${\bar{H}}^{\text{ZQ}}$ along the $I_{z}^{\text{ZQ}}$ axis is obtained, which is according to Eq. ([Disp-formula Ch1.E34]) proportional to the MAS frequency. The effective rotation axis is tilted away from the $I_{x}^{\text{ZQ}}$ direction by an angle ϕ (Eq. 23). The effective rotation frequency $\omega_{\text{eff}}^{\text{ZQ},+1}$ (Eq. 22) increases with increasing mismatch. Likewise, the $I_{x}^{\text{ZQ}}$ component of ${\bar{H}}^{\text{ZQ}}$ decreases with the decreasing effective dipolar coupling. This amplifies the effect of the RF field inhomogeneity on the orientation of the effective Hamiltonian axis. The state vector rotates on the surface of a cone (Fig. 7a, b and d, e). As a consequence, the inversion becomes inefficient. Only the central part of the sample yields a high transfer efficiency.

### 3.3 RF amplitude sweeps in the absence of RF field inhomogeneity

Continuous RF amplitude sweeps are used to improve the cross-polarization efficiency. In this case, the effective Hamiltonian changes its orientation in the course of the pulse sequence. An adiabatic inversion is achieved if two conditions are fulfilled: (i) the initial state vector is aligned with the initial effective field vector and (ii) the effective field changes its orientation slowly. We focus on the zero-quantum ( n=+1 ) condition assuming a dipolar coupling constant $b_{\text{IS}}$ = 10 kHz . In the following, spin state trajectories are calculated for two sweep amplitudes: Δ = 10 and 30 kHz .

**Figure 8:**
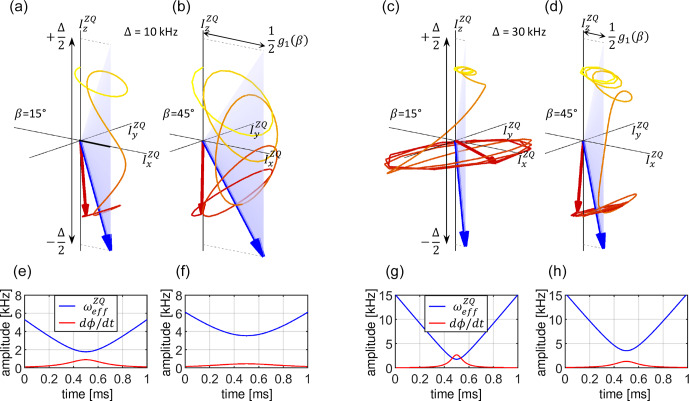
Visualization of the spin state trajectories for the linear ramp cross-polarization experiment assuming a homogeneous RF field distribution. For the simulation, a dipolar coupling $b_{\text{IS}}$ = 10 kHz was assumed. The CP contact time was set to T = 1 ms . The calculation was carried out for two crystallite orientations ( β = 15 and 45 ${}^{\circ }$ ; panels **a, c** and **b, d**, respectively) and two sweep amplitudes ( Δ = 10 kHz and Δ = 30 kHz ; panels **a, b** and **c, d**, respectively). The blue-shaded areas represent the changing effective Hamiltonian. The blue arrow indicates the effective Hamiltonian at the end of the pulse sequence at t=T . The component along the $I_{z}^{\text{ZQ}}$ axis is time dependent, while the $I_{x}^{\text{ZQ}}$ axis component is fixed (see Eq. [Disp-formula Ch1.E40]). The beginning of the trajectory is depicted as a yellow line which gradually turns red as the trajectory progresses. The final state of the spin state vector (initially oriented along $I_{z}^{\text{ZQ}}$ ) is drawn as a red arrow. Panels **(e–h)** display dϕ(t)/dt and $\omega_{\text{eff}}^{\text{ZQ}}(t)$ . In **(c, g)**, the adiabaticity condition $d\phi /dt<\omega_{\text{eff}}$ is violated during the sweep.

The spin state trajectories for the linear ramp are represented in Fig. 8. The $I_{x}^{\text{ZQ}}$ component of the effective Hamiltonian is fixed in time and is given by the effective dipolar coupling at a given orientation (Eq. [Disp-formula Ch1.E40], assuming ξ = 1.0). The maximal value of $\frac{1}{2}g_{1}(\beta )$ is reached for β = 45 ${}^{\circ }$ , which together with the sweep amplitude of Δ = 10 kHz and according to Eq. (23) results in a tilt angle of the effective field $\phi (t=0,\beta =45{}^{\circ })$ = 54.7 ${}^{\circ }$ at the beginning of the pulse sequence (Fig. 8b). Clearly, the initial state vector $\rho^{\text{ZQ}}(0)=I_{z}^{\text{ZQ}}$ is not aligned with the effective field of ${\bar{H}}^{\text{ZQ}}(t=0)$ . However, the inversion efficiency is high due to the slow change in the orientation of the effective field, dϕ/dt , such that the state vector can follow the effective field while it is rotating around it in rather large circles (see evaluation of the adiabaticity condition in Fig. 8f). For a smaller effective dipolar coupling (for example, β = 15 ${}^{\circ }$ in Fig. 8a), the angle ϕ is larger (close to 90 ${}^{\circ }$ ). During the linear ramp, the effective Hamiltonian amplitude $\omega_{\text{eff}}^{\text{ZQ}}(t)$ goes through a minimum in the middle of the sweep at t=T/2 , where its value is solely determined by the effective dipolar coupling; see Eq. (22). At the same time, dϕ/dt reaches its maximum (Fig. 8e). Under these conditions, the state vector keeps track of the effective field (Fig. 8a). When a larger sweep amplitude is employed, e.g., Δ = 30 kHz , the orientation of the initial effective field is closer to the $I_{z}^{\text{ZQ}}$ axis, $\phi (t=0,\beta =45{}^{\circ })$ = 76.7 ${}^{\circ }$ (Fig. 8d). At the same time, the amplitude of the effective Hamiltonian $\omega_{\text{eff}}^{\text{ZQ}}(t=0)$ is increased as well. For the crystallite orientation β = 15 ${}^{\circ }$ (Fig. 8c), however, we find that the adiabaticity condition is violated in the middle of the pulse sequence (Fig. 8g). The state vector is not able to follow the effective field as dϕ/dt becomes too high. As a consequence, the state vector keeps rotating near the Equator (Fig. 8c) and thus contributes little to the total transfer efficiency.

**Figure 9:**
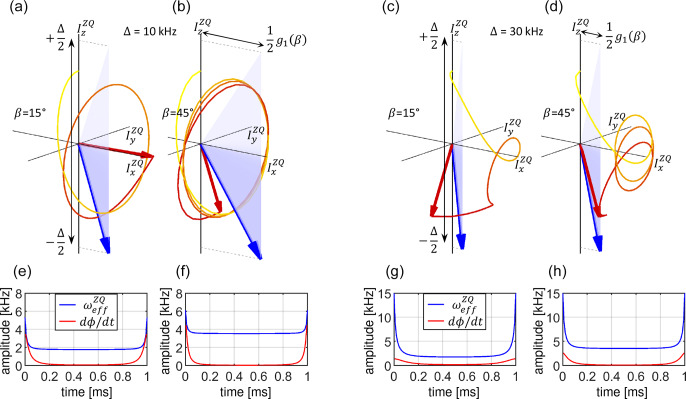
Visualization of the spin state trajectories for the adiabatic tangential sweep cross-polarization experiment assuming a homogeneous RF field distribution. For the simulation, a dipolar coupling $b_{\text{IS}}$ = 10 kHz was assumed. The CP contact time was set to T = 1 ms . The calculation was carried out for two crystallite orientations ( β = 15 and 45 ${}^{\circ }$ ; panels **a, c** and **b, d**, respectively) and two sweep amplitudes ( Δ = 10 kHz and Δ = 30 kHz ; panels **a, b** and **c, d**, respectively, b=Δ/50) . The blue-shaded areas represent the changing effective Hamiltonian. The blue arrow indicates the effective Hamiltonian at the end of the pulse sequence at t=T . The component along the $I_{z}^{\text{ZQ}}$ axis is time dependent, while the $I_{x}^{\text{ZQ}}$ axis component is fixed (see Eq. [Disp-formula Ch1.E40]). The beginning of the trajectory is depicted as a yellow line which gradually turns red as the trajectory progresses. The final state of the spin state vector (initially oriented along $I_{z}^{\text{ZQ}}$ ) is drawn as a red arrow. Panels **(e–h)** display dϕ(t)/dt and $\omega_{\text{eff}}^{\text{ZQ}}(t)$ . In **(a, e)** and **(b, f)**, the adiabaticity condition dϕ/dt < $\omega_{\text{eff}}$ is violated during the sweep.

Spin state trajectories for the adiabatic variant of the CP experiment are shown in Fig. 9. The tangential sweep has been suggested to keep the rate of change dϕ(t)/dt small compared to the effective field amplitude at all times (Hediger et al., 1995). Initially, $\omega_{\text{eff}}(t)$ is large implying that dϕ(t)/dt can be large. However, for small sweep amplitudes such as Δ = 10 kHz , the effective field changes too rapidly for a portion of crystallites at the beginning and at the end of the sweep so that the adiabaticity condition is violated (Fig. 9e). Most of the dynamics take place when the tangential function goes through the central plateau, where the RF amplitudes do not change significantly over an extended period of time. The state vector rotates in large circles around the effective Hamiltonian that is oriented predominantly along the $I_{x}^{\text{ZQ}}$ axis. When a larger sweep amplitude Δ = 30 kHz is used, the adiabatic regime is restored for most crystallite orientations and an improved transfer efficiency is obtained.

**Figure 10:**
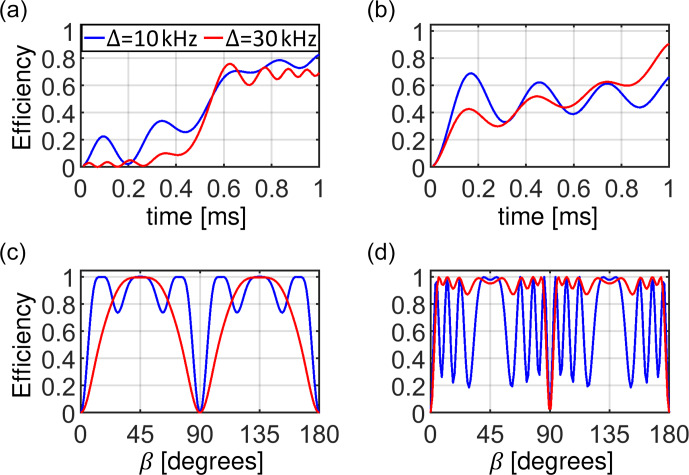
Powder-averaged buildup of the transferred magnetization during the mixing time of the CP experiment **(a, b)** and the final transfer efficiency as a function of crystallite orientation **(c, d)** for an RF amplitude sweep using a linear ramp **(a, c)** and a tangential shape **(b, d)**. The blue and red curves correspond to sweep amplitudes of 10 and 30 kHz , respectively. In all simulations, a homogeneous RF field distribution is assumed.

Figure 10 compares the magnetization transfer during the RF sweep for the examples discussed above. The transfer process is fast when the change in the effective field orientation is fast: in the middle of the linear ramp and at the beginning and at the end of the tangential sweep, provided the adiabaticity condition is maintained (Fig. 10a and b). Figure 10c and d shows the transfer efficiency as a function of crystallite orientation. Note that the spin state inversion cannot be achieved for crystallite orientations with an effective dipolar coupling that is vanishing, i.e., for β = 0 and 90 ${}^{\circ }$ . The portion of crystallites yielding low transfer depends on the ratio of the sweep amplitude Δ and the dipolar coupling $b_{\text{IS}}$ . For the linear ramp, Δ = 10 kHz is preferable, while the tangential sweep using an amplitude Δ = 30 kHz yields high efficiency for most of the crystallites under the conditions investigated here. After powder averaging, the magnetization transfer efficiency is on the order of 90 % for the tangential sweep. We would like to note that all predictions based on the ZQ average Hamiltonian agree well with exact simulations using SIMPSON.

**Figure 11:**
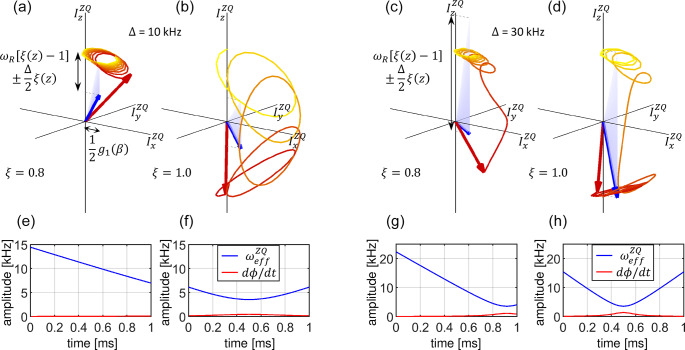
Visualization of the spin state trajectory for a linear ramp cross-polarization experiment assuming an inhomogeneous RF field distribution. For the simulation, a dipolar coupling $b_{\text{IS}}$ = 10 kHz was assumed. The CP contact time was set to T = 1 ms . The calculation was carried out for one crystallite orientation ( β = 45 ${}^{\circ }$ ) and two positions along the coil axis with RF field scaling factors ξ = 0.8 and 1.0 (panels **a, c** and **b, d**) and two sweep amplitudes Δ = 10 kHz and Δ = 30 kHz (panels **a, b** and **c, d**). The blue-shaded areas represent the changing effective Hamiltonian. The blue arrow indicates the effective Hamiltonian at the end of the pulse sequence at t=T . The component along the $I_{z}^{\text{ZQ}}$ axis is time dependent, while the $I_{x}^{\text{ZQ}}$ axis component is fixed (see Eq. [Disp-formula Ch1.E40]). The beginning of the trajectory is depicted as a yellow line which gradually turns red as the trajectory progresses. The final state of the spin state vector (initially oriented along $I_{z}^{\text{ZQ}}$ ) is drawn as a red arrow. Panels **(e–h)** display dϕ(t)/dt and $\omega_{\text{eff}}^{\text{ZQ}}(t)$ to appreciate whether the adiabaticity condition dϕ/dt < $\omega_{\text{eff}}$ is violated during the sweep.

### 3.4 RF amplitude sweeps in the presence of an inhomogeneous RF field

In the following paragraph, RF field inhomogeneities are included in the analysis. For simplicity, we assume that the RF field varies along the solenoid coil axis as described in Fig. 2 and the variation is the same for both RF channels. We disregard time modulations induced by sample rotation in a spatially inhomogeneous RF field. We assume that the Hartmann–Hahn condition is fulfilled for the nominal RF amplitudes in the middle of the coil. The RF amplitude sweep is applied to the I channel. We again examine the zero-quantum ( n=+1 ) recoupling condition. The drive Hamiltonian ${\bar{H}}^{\text{ZQ}}$ is given by Eq. ([Disp-formula Ch1.E40]). Sweeping the RF amplitude makes the $I_{z}^{\text{ZQ}}$ component of the effective Hamiltonian time dependent. The range over which it varies depends on the position along the coil axis, and it is visualized in Fig. 4. The center of the sweep is shifted away from the exact matching condition towards the ends of the coil by an amount that depends on the MAS frequency. As discussed above, the evolution in the double-quantum subspace can be neglected, since ${\bar{H}}^{\text{DQ}}$ has a dominant component along $I_{z}^{\text{DQ}}$ axis which is much larger than the effective dipolar coupling. This can be achieved by choosing a proper value for $\omega_{S}^{\text{NOM}}$ . At the same time, we have chosen conditions that avoid simultaneous matching of different Hartmann–Hahn conditions within the sample volume.

The previous description of the RF-amplitude-modulated CP is valid at the center of the coil where ξ = 1.0. The situation is quite different in volume elements towards the ends of the coil. Figure 11 illustrates the spin state trajectories for the linear ramp CP experiment, assuming a crystallite angle β = 45 ${}^{\circ }$ , a MAS frequency of $\omega_{R}/2\pi$ = 50 kHz and a dipolar coupling constant of $b_{\text{IS}}$ = 10 kHz . The scaling factor ξ = 0.8 is realized for z=±0.36l (where l is the coil length) around the center of the coil. When the sweep amplitude is Δ = 10 kHz , the effective field does not get inverted during the sweep (Figs. 4a and 11a) and therefore cannot invert the spin state, regardless of its adiabaticity (Fig. 11e). Increasing the sweep amplitude to Δ = 30 kHz yields better results as the effective field approaches the Hartmann–Hahn recoupling condition towards the end of the sweep period (Figs. 4b and 11c).

**Figure 12:**
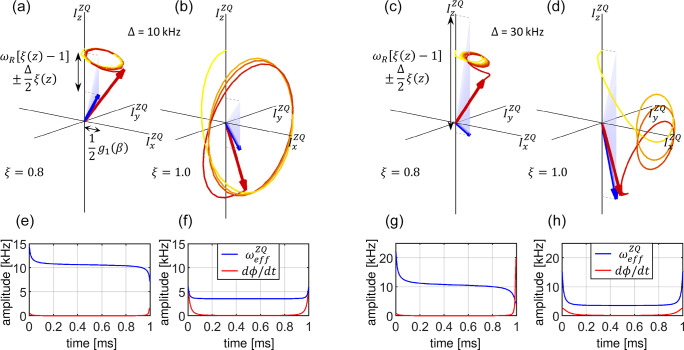
Visualization of the spin state trajectory for an adiabatic tangential sweep cross-polarization experiment assuming an inhomogeneous RF field. For the simulation, a dipolar coupling $b_{\text{IS}}$ = 10 kHz was assumed. The CP contact time was set to T = 1 ms . The calculation was carried out for one crystallite orientation ( β = 45 ${}^{\circ }$ ) and two positions along the coil axis with RF field scaling factors ξ = 0.8 and 1.0 (panels **a, c** and **b, d**) and two sweep amplitudes Δ = 10 kHz and Δ = 30 kHz , assuming b=Δ/50 (panels **a, b** and **c, d**). The blue-shaded areas represent the changing effective Hamiltonian. The blue arrow indicates the effective Hamiltonian at the end of the pulse sequence at t=T . The component along the $I_{z}^{\text{ZQ}}$ axis is time dependent, while the $I_{x}^{\text{ZQ}}$ axis component is fixed (see Eq. [Disp-formula Ch1.E40]). The beginning of the trajectory is depicted as a yellow line which gradually turns red as the trajectory progresses. The final state of the spin state vector (initially oriented along $I_{z}^{\text{ZQ}}$ ) is drawn as a red arrow. Panels **(e–h)** display dϕ(t)/dt and $\omega_{\text{eff}}^{\text{ZQ}}(t)$ to appreciate whether the adiabaticity condition dϕ/dt < $\omega_{\text{eff}}$ is violated during the sweep.

For a tangential sweep, the spin state trajectories are depicted in Fig. 12. Initially, and towards the end of the sweeping period, the RF amplitude changes rapidly and so does the effective field orientation. This can lead to a violation of the adiabaticity condition, as encountered for the calculation with a sweep amplitude of Δ = 30 kHz (Fig. 12c and g). Despite the fact that the Hartmann–Hahn matching condition is included within the sweep range, the state vector does not follow the effective field. These parts of the sample yield a low transfer efficiency.

**Figure 13:**
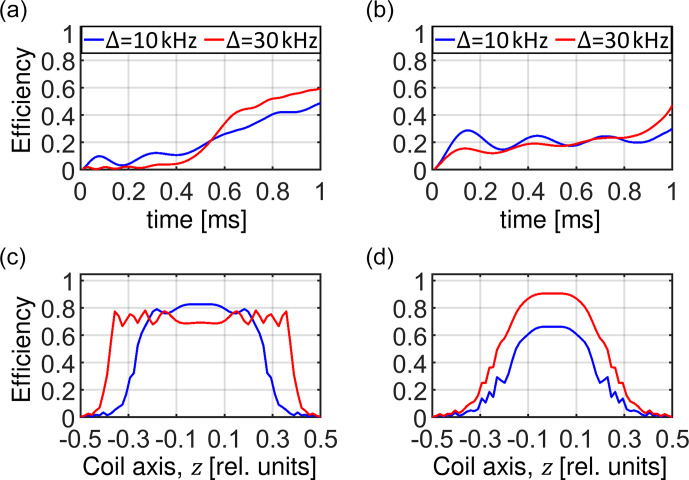
Powder-averaged buildup of transferred magnetization during the mixing time of the CP experiment **(a, b)** and the final powder-averaged transfer efficiency as a function of the position along the coil axis **(c, d)** for a linear ramp **(a, c)** and a tangential shape **(b, d)**. The blue and red curves correspond to sweep amplitudes of Δ = 10 kHz and Δ = 30 kHz , respectively. In the calculation, an inhomogeneous RF field is assumed.

The buildup of the transferred magnetization integrated over the sample volume and detected by the NMR coil for both the linear ramp and the tangential sweep is presented in Fig. 13a and b. It is not obvious which sweeping method will yield a higher total transfer efficiency. Of the four setups discussed so far, the linear ramp with Δ = 30 kHz yields the best result. When comparing efficiency profiles along the coil axis (Fig. 13c and d), we observe that a tangential sweep is more efficient near the center of the coil but quickly loses efficiency when going towards the ends. However, a linear ramp yields equal transfer over a larger sample volume.

**Figure 14:**
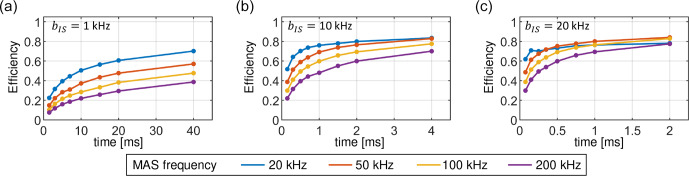
Maximum achievable transfer efficiencies in the cross-polarization experiment as a function of contact time and MAS frequency using numerical optimizations. Similar efficiencies are obtained for both the linear ramp and the tangential sweep, although different shape parameters have to be employed. Dipolar couplings of 1, 10 and 20 kHz are used in the simulations for panels **(a–c)**, respectively.

**Figure 15:**
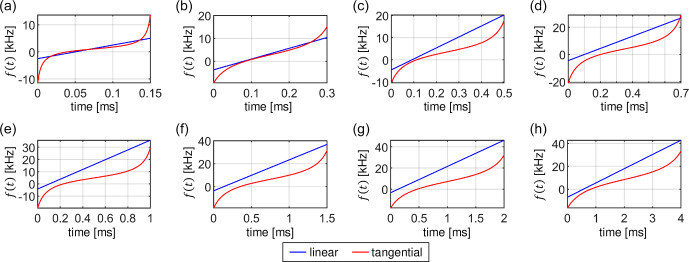
Comparison of optimal linear ramp (blue) and tangential sweep (red) shapes obtained by numerical optimizations at different contact times T = 0.15, 0.3, 0.5, 0.7, 1.0, 1.5, 2.0 and 4.0 ms in panels **(a–h)**, respectively. For the optimization, a dipolar coupling $b_{\text{IS}}$ = 10 kHz was assumed. The calculations were performed assuming a MAS frequency of 50 kHz and a realistic RF inhomogeneity distribution. Although the two shapes are different, they yield virtually identical total transfer efficiencies.

### 3.5 Numerical optimizations of linear and tangential sweeps

In this section, we discuss which parameters of a linear ramp and a tangential sweep yield the best transfer efficiency. We address this problem by a numerical optimization. The calculations are repeated for a range of dipolar couplings and MAS frequencies. In the case of the linear ramp, the sweep amplitude Δ and the offset $\delta_{M}$ from the exact Hartmann–Hahn condition are optimized. In the case of the tangential sweep, the curvature parameter b is considered in addition (Fig. 3). The offset parameter $\delta_{M}$ corresponds to the mismatch of the recoupling condition in the middle of the coil due to RF inhomogeneity and reflects the experimental optimization procedure where the amplitude $\omega_{S}^{\text{NOM}}$ is kept constant and the amplitude $\omega_{I}^{0}$ is optimized around the expected recoupling condition. To ensure that no more than one matching condition is encountered during the sweep, the amplitude Δ was restricted to values within ± $\omega_{R}/2$ (Hediger et al., 1995). The dynamics were evaluated using the effective Hamiltonian ${\bar{H}}^{\text{ZQ}}$ given in Eq. ([Disp-formula Ch1.E40]). The optimized parameters correspond to the best transfer efficiency obtained from 100 repetitions initiated by random guess. As expected, we obtain a different set of optimal parameters for each contact time, dipolar coupling and MAS frequency.

The optimized transfer efficiencies are summarized in Fig. 14. Remarkably, we have not found any significant differences in the performance of the linear ramp with respect to the tangential sweep. Both sweep methods yield the same total transfer efficiency, although they use different sweep parameters. An example of the best sweep shapes obtained for a dipolar coupling $b_{\text{IS}}$ = 10 kHz and a MAS frequency of 50 kHz is presented in Fig. 15. The tangential sweeps tend to have a larger sweep amplitude Δ and a smaller offset values $\delta_{M}$ when compared to the linear ramp.

We observe that very long contact times are required to obtain high transfer efficiencies. For calculations involving different dipolar coupling strengths $b_{\text{IS}}$ the same range of the reduced time parameter $Tb_{\text{IS}}$ is used. In this way, longer mixing times T are maintained for smaller dipolar couplings $b_{\text{IS}}$ . Better performance is obtained for cases with higher dipolar couplings, which correlates with the width of Hartmann–Hahn conditions in CP matching profiles. On the other hand, the transfer efficiency decreases at higher MAS frequencies due to increased volume selectivity. Small dipolar couplings are the most challenging, on the order of 1 kHz , and ultrafast MAS ( > 100 kHz ), which are typical of ${}^{15}N$ – ${}^{13}C$ spin pairs in proteins studied by proton-detected MAS solid-state NMR experiments. To more efficiently average proton dipolar interaction, MAS probe development aims at smaller-diameter rotors to achieve higher MAS rotation frequencies. Currently, 0.4 mm MAS probes are in development that can reach MAS frequencies of up to 200 kHz . Our predictions suggest that only 20 % of the sample will contribute to the detected NMR signal after a 10 ms ${}^{15}N$ – ${}^{13}C$ CP mixing step at a MAS frequency of 200 kHz ; i.e., up to 80 % of the signal is lost in a single magnetization transfer step. The efficiency increases to ca. 40 % when a 40 ms long mixing period is used, provided that there are no signal losses due to relaxation. However, note that the sensitivity in a pulse sequence with multiple CP transfer elements depends on all previous transfer steps. The first CP element preselects a volume that is maintained or further restricted in subsequent transfer elements.

**Figure 16:**
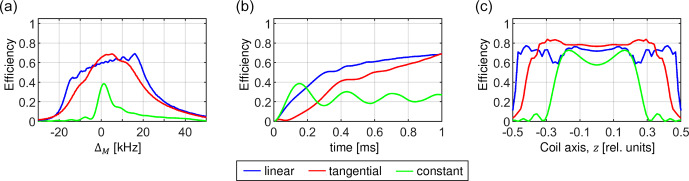
Comparison of the matching profiles **(a)**, magnetization transfer buildups **(b)** and contribution to the transfer efficiency of individual volume elements along the coil axis **(c)** for an optimized linear ramp (blue), a tangential sweep (red) and a constant amplitude CP (green). For the optimization, a dipolar coupling $b_{\text{IS}}$ = 10 kHz was assumed. The calculations were performed assuming a MAS frequency of 50 kHz and a realistic RF inhomogeneity distribution. The CP contact time was set to T = 1 ms . In **(a)** and **(c)**, the constant amplitude CP was evaluated after 160 µs when it reaches maximum transfer efficiency.

We find that there is no difference between the linear ramp and the tangential shapes in terms of total transfer efficiency. In Fig. 16, we compare these two methods (together with a constant amplitude CP) with respect to the width of the CP matching profile (Fig. 16a), the magnetization transfer buildup (Fig. 16b) and the sample volume selectivity (Fig. 16c). As expected, the RF amplitude sweep significantly improves the width and the height of the matching profile. The most important difference is that the tangential sweep yields higher efficiency near the center of the coil and lower efficiency at edges of the coil. Use of RF pulses and other recoupling elements can potentially result in a preselection of a particular sample volume that cannot be utilized by the linear ramp for a further transfer. Therefore, transfer elements should be optimized within the framework of the whole pulse sequence to minimize a differential preselection of the sample volume during calibration experiments.

The linear ramp and the adiabatic tangential sweeps were calculated for the ZQ ( n=+1 ) condition. However, the shapes are equally applicable to any other n = ± 1 Hartmann–Hahn condition, as the corresponding effective Hamiltonian has the same form. The n = ± 2 Hartmann–Hahn conditions suffer from increased RF field inhomogeneity (factor of 2 in Eq. 40) and have different powder averaging properties implied by the $g_{2}(\beta )$ term. Thus, a decreased CP transfer efficiency for the n = ± 2 matching condition is expected.

The transfer efficiencies of all pulse sequences were verified using numerical simulations in SIMPSON. To avoid overlap of the different Hartmann–Hahn matching conditions, the zero-quantum ( n=+1 ) condition with $\omega_{S}^{\text{NOM}}/2\pi$ = 60 kHz was selected using MAS frequencies of 20 and 50 kHz , while the double-quantum ( n=+1 ) condition with $\omega_{S}/2\pi$ = 30 kHz was used for a MAS frequency of 100 kHz . The agreement between SIMPSON and the effective Hamiltonian calculations is excellent except for a simulation in which a dipolar coupling of 20 kHz and a MAS frequency of 20 kHz was assumed. In this case, the numerically evaluated transfer efficiencies are about 10 % lower. A plausible explanation is that the first-order average Hamiltonian approximation does not provide the full description of the spin dynamics when the dipolar coupling and the MAS frequency are of similar value (in other cases it holds $b_{\text{IS}}\ll \omega_{R}/2\pi )$ .

## 4 Conclusions

We have analyzed the magnetization transfer efficiency of the CP experiment as a function of the MAS frequency in the presence of RF field inhomogeneity of a solenoid coil. We show that a sweep of the RF amplitude through the Hartmann–Hahn matching conditions using either a linear ramp or a tangential shape improves the performance in a comparable way. We do not observe a difference in the total transfer efficiency between these two methods. We find that magnetization transfer using a CP recoupling element becomes inefficient in particular for small dipolar couplings for ultrafast MAS experiments with rotation frequencies above 100 kHz . New recoupling methods that are designed explicitly to account for inhomogeneous RF fields and ultrafast MAS conditions are needed to overcome this issue in the future.

## Data Availability

No data sets were used in this article.
